# Knockdown of CD44 inhibits the invasion and metastasis of hepatocellular carcinoma both *in vitro* and *in vivo* by reversing epithelial-mesenchymal transition

**DOI:** 10.18632/oncotarget.3488

**Published:** 2015-03-08

**Authors:** Yuan Gao, Bai Ruan, Weihui Liu, Jianlin Wang, Xisheng Yang, Zhuochao Zhang, Xia Li, Juanli Duan, Fuqing Zhang, Rui Ding, Kaishan Tao, Kefeng Dou

**Affiliations:** ^1^ Department of Hepato-Biliary and Pancreto-Splenic Surgery, Xijing Hospital, The Fourth Military Medical University, Xi'an, China; ^2^ General Surgery Center of PLA, Chengdu Military General Hospital, Chengdu, China

**Keywords:** CD44, Hepatocellular carcinoma, epithelial-mesenchymal-transition, Snail

## Abstract

Mounting evidence has shown that induction of epithelial-mesenchymal transition (EMT) contributes to the the expression of CSC (cancer stem cell) markers. However, whether and how CSC markers could be involved in regulating EMT has rarely been reported. CD44, being one of the most commonly used CSC markers in hepatocellular carcinoma (HCC), has been demonstrated to act as a multidomain, transmembrane platform that serves to integrate a wide variety of extracellular signals. Therefore, we determined to seek whether CD44 is necessary for the EMT process in HCC. First, we noticed that CD44 expression was associated with the mesenchymal phenotype in HCC cell lines, and knocking down CD44 with lentivirus-mediated shRNA in HCC cell lines resulted in the mesenchymal-epithelial-transition (MET) and the subsequent impaired migration and invasion *in vitro*. Moreover, in a metastatic mice model established by tail vein injection of luciferase labelled MHCC97-H cells, we confirmed that CD44 knockdown resulted in the decreased metastasis of HCC cells. Furthermore, we found that the induction of MET by CD44 inhibition might be achieved, at least in part, by repressing the ERK/Snail pathway.

## INTRODUCTION

Liver cancer is the sixth most prevalent cancer [1] and the second most common cause of cancer death worldwide [2], and hepatocellular carcinoma (HCC) is the major histological subtype of liver cancer. Though there are many treatment options for HCC patients, including surgical resection, ablation, chemoembolization, liver transplantation, and sorafenib, the prognosis for HCC remains poor, which is mainly because of the high level of tumor invasiveness, frequent intrahepatic spread and extrahepatic metastasis [3].

The epithelial–mesenchymal transition (EMT) has been demonstrated to promote cell invasion, leading to tumor cell metastasis in various cancers, including HCC [4-6]. During the EMT, epithelial cells lose their junctions and apical-basal polarity, acquire the motility and the invasiveness properties of mesenchymal cells characterized by the down-regulation of cell-cell adhesion molecules, such as E-cadherin, claudins and occludin and the up-regulation of vimentin, fibronectin and N-cadherin [7]. Conversely, the mesenchymal-epithelial transition (MET) describes the reverse process. Transcription factors such as Twist1, ZEB2, Snail and FOXC2 can directly or indirectly trigger the EMT [8-10]. However, the mechanism of how these switch factors are regulated in different conditions merits further study.

Recently, powerful studies showed that the induction of the EMT generates both normal and cancer cells with stem cell properties and results in the overexpression of stem cell markers [11-13]. However, it is unclear whether the altered expression of cancer stem cell (CSC) markers would conversely affect the EMT process. CD44, one of the most commonly used CSC markers [14-18], has been reported to be significantly correlated with the presence of vascular invasion and the poor prognosis of HCC [19, 20]. And the CD44^+^ CSCs are supposed to be responsible for the long-term maintenance of tumor growth, metastasis and chemoresistance [21, 22]. What's more, as a major adhesion molecule of the extracellular matrix, CD44 was demonstrated to be a multidomain, transmembrane platform that serves to integrate a wide variety of cell-surface receptor tyrosine kinases (RTKs) [23], whose ligation to growth factors were demonstrated to be able to induce partial or full EMT [8].

In the present study on HCC, we explored the relationship between CD44 expression and EMT phenotypes, and investigated the impact of CD44 knockdown on the EMT and the subsequent invasion and metastasis of HCC cells both *in vitro* and *in vivo*. Furthermore, we found that the MET and subsequent impaired invasion and metastasis of HCC cells by CD44 knockdown are at least in part, might due to the repression of the ERK/Snail pathway.

## RESULTS

### CD44 expression was associated with the mesenchymal phenotype in HCC cells

To gain insight into the relationship between CD44 and the mesenchymal phenotype in HCC cells, we examined the mRNA, protein expression of CD44 and EMT representative markers (E-cadherin and N-cadherin) in 4 different HCC cell lines (Huh7, SMMC-7721, MHCC97-H and HepG2), which have different invasion capacities, according to a study from our group [24]. As consistently shown by qRT-PCR and Western blot, CD44 was abundantly expressed in mesenchymal SMMC-7721 and MHCC97-H cells, which have higher E-cadherin and lower N-cadherin than the epithelial Huh7 and HepG2 cells, indicating that the CD44 level was associated with maintenance of the mesenchymal phenotype in HCC cell lines.

**Fig.1:**
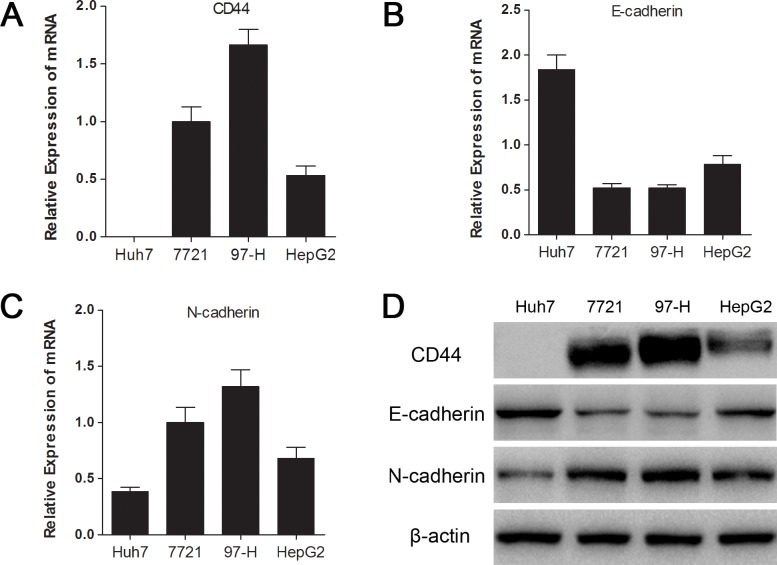
CD44 expression was associated with the mesenchymal phenotype in HCC cell lines The relative mRNA expressions of CD44 (A), E-cadherin (B) and N-cadherin (C) in 4 HCC cell lines are separately presented as histograms. (D) The CD44, E-cadherin, and N-cadherin protein levels were determined by Western blot analysis. β-actin was used as an internal control.

### Knockdown of CD44 induced MET in SMMC-7721 and MHCC97-H cells

**Fig.2:**
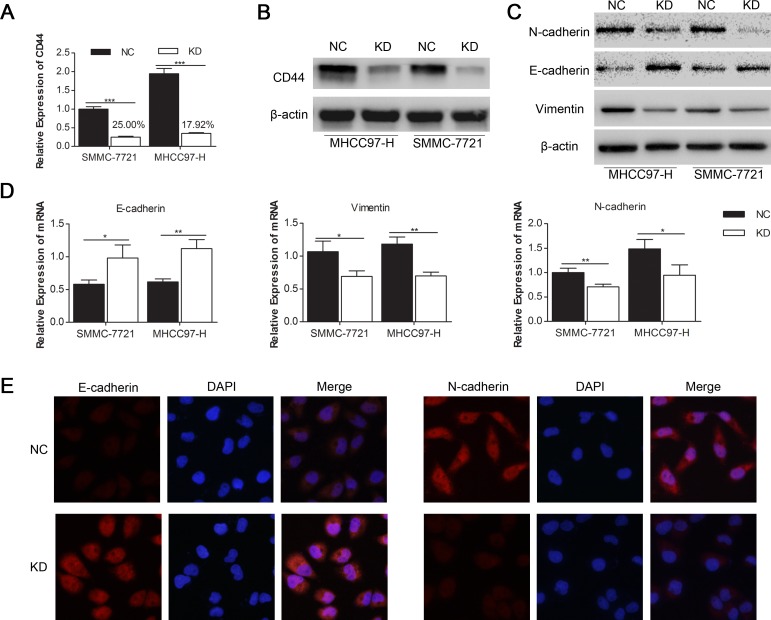
Knockdown of CD44 induced the MET in SMMC-7721 and MHCC97-H cells qRT-PCR (A) and Western-blot (B) analysis of CD44 expression in SMMC-7721 and MHCC97-H cells that were transfected with shRNA of CD44 (KD) and normal control (NC). The expression levels of proteins (C) and mRNA (D) of the EMT markers are shown. (E) The protein expression levels of E-cadherin and N-cadherin in SMMC-7721 cells transfected with shRNA of CD44 and NC are shown by immunofluorescences under a 400× field. (* means p<0.05, ** means p<0.01, *** means p<0.001 by T-test).

To investigate the influence of CD44 knockdown on the EMT in HCC cells, we transfected lentivirus with shRNA of CD44 or NC into SMMC-7721 and MHCC97-H cells respectively, and got the stably passaged SMMC-7721-KD, SMMC-7721-NC, MHCC97-H-KD and MHCC97-H-NC. qRT-PCR showed a dramatic CD44 mRNA decrease of 75.01% in SMMC-7721 and 82.08% in MHCC97-H with lentivirus transfection (Fig. 2A). Western blot confirmed the inhibition of CD44 protein in both SMMC-7721 and MHCC97-H cells (Fig. 2B). Because of the CD44 knockdown, the enhanced expression of epithelial marker (E-cadherin) and the reduced expression of mesenchymal markers (N-cadherin and Vimentin) in MHCC97-H-KD and SMMC-7721-KD cells were detected by Western blot (Fig. 2C), qRT-PCR (Fig. 2D) and immunofluorescence (Fig. 2E). These results demonstrated that the knockdown of CD44 in SMMC-7721 and MHCC97-H cells induced a transition to the epithelial phenotype.

### CD44 knockdown inhibited migration and invasion in MHCC97-H and SMMC-7721 cells

**Fig.3:**
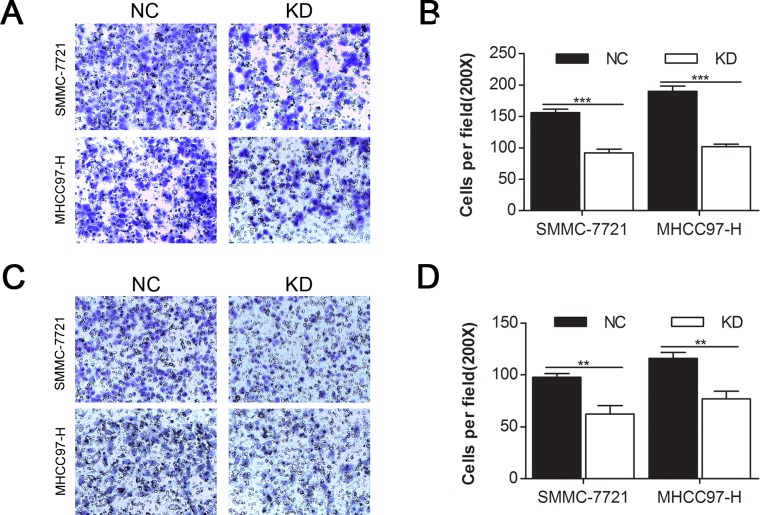
CD44 knockdown inhibited the migration and invasion of MHCC97-H and SMMC-7721 cells Migration (A) and matrigel invasion (C) assays of SMMC-7721 and MHCC97-H cells that were transfected with shRNA of CD44 or NC were evaluated. Migrated (B) and invaded (D) cells were counted under 3 randomised 200× field (* means p<0.05, ** means p<0.01, *** means p<0.001 by T test).

As cell migration and invasion properties are important consequences of the EMT [25, 26], we investigated the impact of CD44 knockdown on the migration (Fig. 3A) and invasion (Fig. 3C) of SMMC-7721 and MHCC97-H cells with transwell migration and invasion assays. We found that the migration rate of SMMC-7721-KD and MHCC97-H-KD cells decreased by 41.03% and 46.31% (Fig. 3B) and that the invasion rate of SMMC-7721-KD and MHCC97-H-KD cells decreased by 36.18% and 33.62% (Fig. 3D), compared with SMMC-7721-NC and MHCC97-H-NC cells, respectively.

### CD44 knockdown in luciferase-labeled MHCC97-H cells inhibited lung metastasis in nude mice

**Fig.4:**
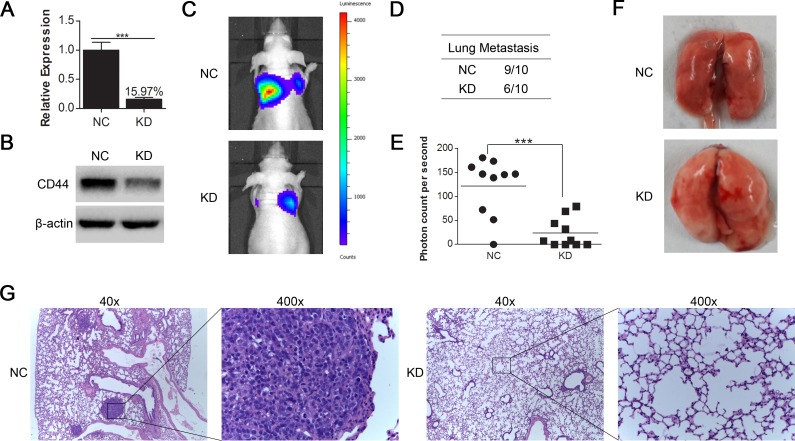
CD44 knockdown in luciferase-labeled MHCC97-H cells inhibited lung metastasis generated by tail vein injection in nude mice (A) qRT-PCR and (B) Western-blot analysis of CD44 expression in luciferase-labeled MHCC97-H cells that were transfected with shRNA of CD44 (KD) or NC. (C) The 10 nude mice in each group were luminescently imaged 45 days after injection (Supplemental Figure 1), and representative images for each group are shown. (D) There was a higher incidence of lung metastases in the CD44 knockdown group than in the normal control group. (E) To quantitively compare the mass of the metastasis, the photon counts per second in each group are shown (*** means p<0.001 by t-test). (F) The lungs of the nude mice from each group were removed and sectioned for evaluation lung metastasis after H&E staining (G).

We transfected the luciferase-labeled MHCC97-H cells with lentivirus containing shRNA of CD44 and normal control as for SMMC-7721 and MHCC97-H cells. The knockdown efficiency of CD44 mRNA was 84.03% (Fig. 4A), which was verified by Western blot (Fig. 4B). The bioluminescent imaging of nude mice 45 days after injection showed that the incidence of lung metastasis was lower in mice injected with MHCC97-H-luc-KD compared with MHCC97-H-luc-NC (Fig. 4D) and that the bioluminescence from MHCC97-H-luc-NC metastases was stronger than that of MHCC97-H-luc-KD (Fig. 4C, E), revealing that the knockdown of CD44 in MHCC97-H-luc cells inhibited lung metastasis in nude mice.

### Knockdown of CD44 induced transition towards epithelial phenotype by repressing ERK/Snail pathway in SMMC-7721 and MHCC97-H cells

**Fig.5:**
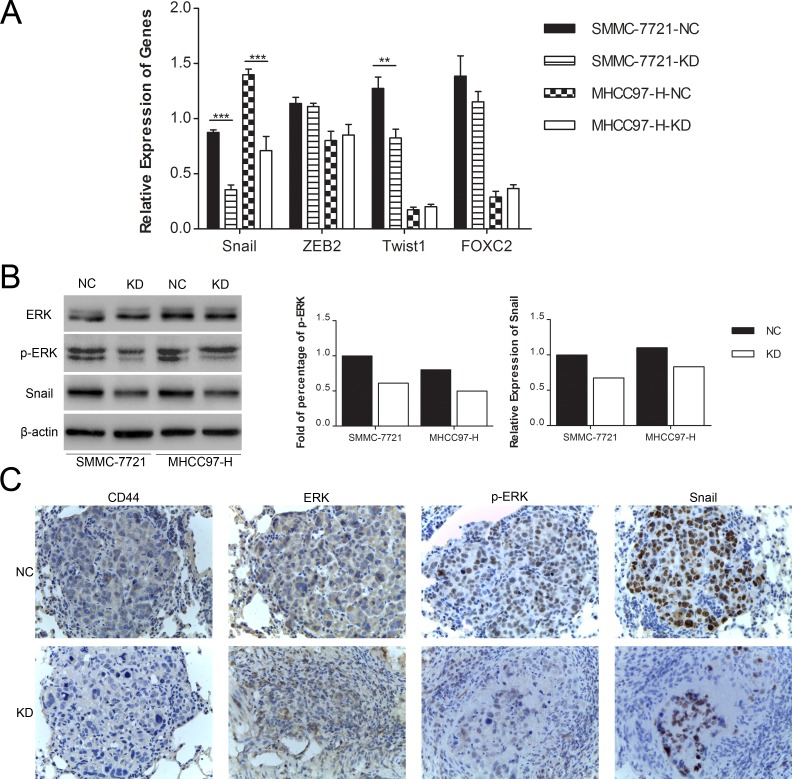
Knockdown of CD44 induced the MET in SMMC-7721 and MHCC97-H cells by repressing the ERK/Snail pathway (A) qRT-PCR analysis of the mRNA expression of transcription factors that predominantly drive EMT in SMMC-7721 and MHCC97-H cells transfected with shRNA of CD44 or NC. (B) Quantification of Western blot showed different phospho-ERK and Snail expression in SMMC-7721 and MHCC97-H cells with or without CD44-knockdown. (C) The expression of CD44, ERK, p-ERK and Snail was detected in metastatic lung samples by immunohistochemical staining and shown with a 400× field.

Because the hallmark of the EMT is directly or indirectly modulated by several predominant transcription factors, including Snail, Twist1, ZEB2 and FOXC2 [8-10, 27-29], we searched for the possible transcription factors that are regulated by CD44. Among these screened transcription factors, we found that both the mRNA (Fig. 5A) and protein (Fig. 5B) expression of Snail were markedly decreased in both SMMC-7721 and MHCC97-H cells in the KD group compared to their corresponding NC cells. At the mean time there were no significant or consistent changes of ZEB2, Twist1 and FOXC2 mRNA in SMMC-7721 and MHCC97-H cells (Fig. 5A). Because CD44 has been demonstrated to integrate the signaling of RTKs [23], which activates the Ras/Raf/MEK/ERK signaling cascade to phosphorylate ERK [30, 31], and mounting evidence indicated that activation of ERK positively regulates the expression of Snail [32-35], we investigated whether the knockdown of CD44 repressed Snail by inhibiting ERK phosphorylation. Western blot analysis showed that the phosphorylation of ERK1/2 (Thr202/Tyr204) was strongly inhibited in SMMC-7721-KD and MHCC97-H-KD cells, unlike in SMMC-7721-NC and MHCC97-H-NC cells cultured under normal conditions, whereas the total ERK1/2 remained unchanged (Fig. 5B). Moreover, immunohistochemical analysis of the metastatic nodule in nude mice showed decreased ERK phosphorylation (Thr202/Tyr204) and Snail expression in response to CD44 knockdown (Fig. 5C). Taken together, these data indicated that CD44 knockdown might induce the MET in SMMC-7721 and MHCC97-H cells by inhibiting the ERK/Snail pathway, at least in part.

## DISCUSSION

Recently, mounting evidence has shown a direct link between the EMT and stem cell properties, which both powerfully contribute to cancer invasion and metastasis. Additionally, Chaffer and Weinberg [36] suggested a model of cancer metastasis in which the EMT empowers cancer cells to prepare for dissemination from primary tumors and induces non-CSCs to enter a CSC-like state to travel through the circulation system because of their anchorage-independent survival. Moreover, it was confirmed that the induction of the EMT in cancer cells results in the expression of CSC markers [11-13]. Because CSCs and the EMT play such an important role in the invasion and metastasis of HCC, determining whether and how CSC marker regulates the process of EMT should help clarify the relationship between the CSCs and EMT.

As one of the most acknowledged CSC markers in HCC, CD44 is associated with a poor prognosis in HCC patients. Hou et al [37] found that accompanied with CD133, CD44 could define a subgroup of HCC cells that were responsible for the hematogenous metastasis of liver cancers. In addition, a study [38] of breast cancer reported that the EMT traits parallel the CSC phenotype in which CD44 is highly expressed. What's more, ADT (androgen deprivation therapy) in prostate cancer could promote the EMT with increased CD44^+^ stem-like cells [39]. In accordance, CD44^high^ALDH^high^ subset of lung cancer cells was found expressing higher EMT transcription factors [40], which is in agreement with our present findings. We found that CD44 is abundantly expressed in mesenchymal HCC cell lines but is not highly expressed in their epithelial counterparts. Because the EMT is a predominant event driving cancer dissemination, the role of CD44 in this process merits further investigation. After the knockdown of CD44 in two different cell lines, we observed the MET, which is characterized by higher E-cadherin, lower vimentin and N-cadherin, verifying the close link between CD44 and the EMT. Because the onset of the EMT is involved in the acquisition of invasive and metastatic potential [25, 26], we investigated the influence of CD44 knockdown on the migration and invasion of HCC cells and ultimately found that the migration and invasion abilities of HCC cells were significantly decreased with remarkable knockdown of CD44. Additionally, in the metastatic model established via tail vein injection of luciferase-labeled MHCC97-H cells in nude mice, we demonstrated that the knockdown of CD44 inhibited lung metastasis, indicating a promising strategy for establishing CD44-targeted molecular therapies for HCC.

Following a study [41] reporting that tumor necrosis factor-alpha induces EMT in retinal pigment epithelial cells through activating TGF-beta signaling in a manner that depends on the hyaluronan-CD44-moesin interaction, Mima et al [42] demonstrated that CD44 plays a critical role in the TGF-beta mediated EMT in HCC. Nevertheless, we still lack detailed mechanisms through which CD44 regulates the onset of the EMT. As the hallmark of the EMT, down-regulation of E-cadherin in balance with up-regulation of N-cadherin is directly, or indirectly, modulated by the overexpression of transcription factors like Snail, Twist1, ZEB2 and FOXC2 [8-10, 27-29]. Specifically, Snail proteins trigger EMT during both normal development and neoplastic progression through coordinating the repression of epithelial genes and the induction of mesenchymal genes. Moreover, Snail is a major EMT inducer in HCC, whose overexpression is correlated with the poor prognosis of HCC [43]. Here, in the effects screen of CD44 knockdown acting on these transcription factors, we found that both mRNA and protein expression of Snail was strongly, consistently decreased in SMMC-7721 and MHCC97-H cells after CD44 inhibition, rather than Twist1, ZEB2 and FOXC2, indicating that CD44 knockdown may induce the MET of HCC cells through inhibiting Snail. However, the mechanism by which this may occur remains further investigation.

The ERK pathway has long been established as one of the most important intracellular signaling pathways in the pathogenesis of human carcinoma, whose constitutive activation is a frequent event in human cancer and can lead to multiple changes in the expression of various genes involved in cell cycle regulation, differentiation, proliferation, invasion, metastasis and survival [31]. As the induction of EMT by TGF-beta pathway has been well acknowledged [44], phosphorylation of MPAK/ERK pathway by growth factors also contributes to EMT [8]. And, differently activated ERK by TGF-β in benign and cancer cells has been demonstrated to be the answer to the TGF-β paradox [45]. Furthermore, recent influential studies have shown that the activation of RAS/RAF/MEK/ERK signaling modulates the expression of Snail via MSK1/Elk-1-mediated epigenetic regulation, and thus promotes cell motility and invasive behavior in cancer-associated EMT [32]. Moreover, as a major adhesion molecule of the extracellular matrix, CD44 has been shown to contribute to ERK activation by integrating the activation of Met, PDGFR, EGFR and FGFR at a number of levels, including the control of receptor–ligand interactions, and cross-regulation of intracellular signaling through ERM (ezrin–radixin–moesin) proteins, ankyrin, Tiam and IQGAP [23, 46-48]. Here, in HCC cells, we found that Snail expression was markedly decreased by CD44 knockdown and with the attenuation of ERK phosphorylation. This finding suggests that knockdown of CD44 in HCC cells could induce the MET by repressing the ERK/Snail pathway, revealing a potential connection between CD44 and the EMT. However, the question that how CD44 affects the intracellular Ras/Raf/MEK/ERK cascade still needs further merit. What's more, research has shown that CD44 could be internalized and translocated to the nucleus, where it binds to various promoters, which may also provide us potential clue to understand the roles of CD44 [49].

Taken together, we consistently show that CD44 knockdown inhibited the migration, invasive and capacities of HCC cells by reversing the EMT phenotypes of HCC, which might be due to the repression of ERK/Snail pathway. Our findings not only enrich the current understanding of CD44's regulation of the EMT but also suggest that this molecule plays an important role in both maintaining the mesenchymal phenotype and participating in HCC dissemination, demonstrating the potential for establishing CD44-targeted molecular therapy of HCC.

## METHODS

### Cell culture, antibodies and reagents

Human HCC cell lines MHCC97-H, SMMC-7721, HepG2, and Huh7 were obtained from Shanghai cellular biology center, MHCC97-H cells labeled with luciferase (MHCC97-H-luc) were kindly provided by Prof. Yong Chen and Dr. Yang Cao from our laboratory. Cells were cultured in DMEM (Hyclone, Logan, UT, USA) supplemented with 10% fetal bovine serum (Invitrogen, Carlsbad, CA, USA), penicillin (100 units/ml) and streptomycin (100 mg/ml) and incubated at 37°C with 5% CO_2_. Phosphate-buffered saline (PBS) with 0.25% trypsin and 0.01% EDTA was used for cell harvesting and passage. Antibodies for CD44, Vimentin, N-cadherin, ERK, and p-ERK (Thr202/Ty204) were purchased from Cell Signaling Technologies (Beverly, MA, USA). E-cadherin antibody was purchased from Santa Cruz (Santa Cruz, CA, USA). Antibodies for β-actin and Snail were purchased from Abcam (Cambridge, UK).

### Knockdown of CD44

Lentivirus plasmid containing short hairpin RNA (shRNA) of CD44 (KD) and negative control (NC) were designed and produced by Genechem (Shanghai, China). For the transfection, MHCC97-H and SMMC-7721 cells were seeded in 6-well plates and allowed to attach overnight; then, the culture medium was replaced with transfection enhancing solution with 30 MOI lentivirus and 50 μg/ml polybrene, respectively. After 16 hours of transfection, we replaced the transfection medium with the normal one. Cells were harvested for passage or testing when they occupied 80% of the plate.

### Western blot analysis

Strong RIPA buffer (Beyotime, Shanghai, China) with protease inhibitor (Thermo Scientific, Rockford, IL, USA) and phosphatase inhibitor (Thermo Scientific) was used to extract protein. 2× sodium dodecyl sulfate (SDS) was added to the cell lysates. After polyacrylamide gel electrophoresis, the separated proteins were transferred to nitrocellulose membranes with prestained protein marker (Thermo Scientific). After blocking with tris-buffered saline with Tween 20 (TBST) containing 5% bovine serum albumin, the membranes were incubated with primary antibodies diluted in blocking buffer overnight at 4°C. After being washed with TBST, the membranes were incubated with horseradish peroxidase (HRP)-conjugated secondary antibodies (Abcam) for 1 h at room temperature. Proteins were detected in the ChemiDoc^TM^ XRS+ using the Image Lab^TM^ software (Bio-Rad, Hercules, CA, USA) after three washes with TBST.

### Immunofluorescence

Cells cultured on slides were washed three times with PBS and fixed with 4% paraformaldehyde at 4°C for 30 min. After another wash, cells were permeabilized in PBS with 0.1% Triton X-100 for 15 min at room temperature and then blocked with 10% donkey serum albumin (Jackson Immuno Research Laboratories, PA, USA). Then, cells were incubated with primary antibodies overnight at 4°C, followed by incubation with Alexa 594-conjugated secondary antibody (Jackson Immuno Research Laboratories) for 1 h at room temperature and another incubation with DAPI for 10 min. Slides were mounted with an anti-fluorescence quenching agent and observed under an Olympus fluorescence microscope.

### RNA extraction and qRT-PCR analysis

Total RNAs were extracted from cells using RNAiso Plus (Takara, Dalian, China). Reverse transcription was performed with PrimeScript^TM^ Master Mix (Takara) according to its product manual. Then, the qRT-PCR was performed with SYBR Premix EX Taq^TM^ II (Takara) according to its product manual on the real-time PCR detection system Bio-Rad IQ5 (Bio-Rad). Using β-actin as the reference, the data were analysed with a normalized gene expression method (ddCt) through the iQ5 Optical System Software (Bio-Rad). All measurements were performed in triplicate. The sequences of the primer pairs were as follows: CD44 5′-GCAGTCAACAGTCGAAGAAGG-3′ and 5′-TGTCCTCCACAGCTCCATT-3′; ZEB2 5′-CAAGAGGCGCAAACAAGCC-3′ and 5′-GGTTGGCAATACCGTCATCC-3′; Twist1 5′-GTCCGCAGTCTTACGAGGAG-3′ and 5′-GCTTGAGGGTCTGAATCTTGCT-3′; Snail 5′-TCGGAAGCCTAACTACAGCGA-3′ and 5′-AGATGAGCATTGGCAGCGAG-3′; FOXC2 5′-CCTCCTGGTATCTCAACCACA-3′ and 5′-GAGGGTCGAGTTCTCAATCCC-3′; and β-actin 5′-CATGTACGTTGCTATCCAGGC-3′ and 5′-CTCCTTAATGTCACGCACGAT-3′.

### *In vitro* migration and invasion assay

Chambers (Millipore, Billerica, MA, USA) with or without matrigel (BD Biosciences, San Jose, CA, USA) bedding were placed in 24-well plates. Cells (5×10^4^) that were starved overnight were added to the upper chamber room in 200 μl of serum free medium, respectively, while 500 μl of complete medium to the bottom chamber room. After 24 hours of incubation, cells in the upper room were fixed with 95% ethanol and stained for 30 minutes with 4 g/L crystal violet. Cells on the underside of the chambers were counted under a 200× microscope field after the topside of the filter was wiped.

### Immunohistochemistry

Briefly, tissue samples were fixed in formalin and embedded in paraffin. Sections were dewaxed in xylene and rehydrated through graded alcohols and water, and endogenous peroxidases were inactivated with 3% hydrogen peroxide in PBS, followed by incubation with the primary antibody overnight at 4°C and with the biotinylated secondary antibody at room temperature for 1 hour. Then, the sections were detected with a streptavidin-peroxidase complex.

### Establishment of the metastatic model in nude mice through tail vein injection

Athymic nude mice (BALB/C-nu/nu, 6–8 weeks old, male) were obtained from the Animal Center of Chinese Academy of Science (Shanghai, China) and fed under specific pathogen-free conditions in the laboratory animal center of Fourth Military Medical University (Xi'an, China). All studies involving animals were approved by the Research Animal Care and Use Committee of Fourth Military Medical University. We established a lung metastatic model of HCC through tail vein injection of 1×10^6^ MHCC97-H-luc cells per mice. Twenty-four nude mice were randomly divided into 2 groups: one group was injected with MHCC97-H-luc-NC, and the other group was given MHCC97-H-luc-KD. Forty-five days after injection, mice were anesthetized by inhalation and then intraperitoneally injected with 10 μl/g of D-luciferin (Caliper, Hopkinton, MA, USA). Fifteen minutes later, mice were bioluminescently imaged in an IVIS Lumina II Imaging System (Caliper). Then, mice were sacrificed after anesthesia, and the organs were separated after infusion and fixed in formaldehyde for hematoxylin and eosin (H&E) staining and immunohistochemistry detection.

### Statistical analysis

Data are presented as the mean±standard deviation (SD) from at least three independent experiments. Student's t-test (two tailed) was used to evaluate the two groups with SPSS 17.0 software (San Rafael, CA, USA). Data were considered statistically significant at p < 0.05.

## SUPPLEMENTARY MATERIAL FIGURE


