# Supplementary material for: Knockdown of CD44 inhibits the invasion and metastasis of hepatocellular carcinoma both in vitro and in vivo by reversing epithelial-mesenchymal transition
Source: Oncotarget. 2015 Mar 8;6(10):7828–37. doi: 10.18632/oncotarget.3488 (PMC4480719; doi:10.18632/oncotarget.3488)
Supplement: Supplementary file 1 [file oncotarget-06-7828-s001.pdf]

Knockdown of CD44 inhibits the invasion and metastasis of hepatocellular carcinoma both *in vitro* and *in vivo* by reversing epithelial-mesenchymal transition

Supplementary Material

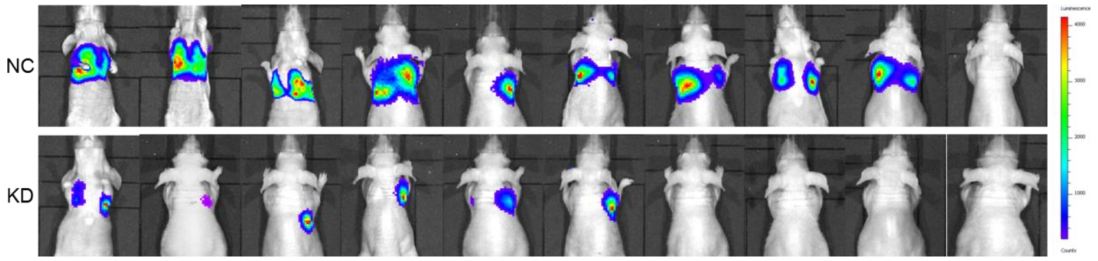

Supplemental Figure 1: The luminescent images of mice injected with luciferase-labeled MHCC97-H. NC means mice injected with MHCC97-H-NC, while KD means mice injected with MHCC97-H-KD.
